# *In situ* ascending aortic thrombus in a patient with metastatic lung adenocarcinoma and no aortic atherosclerosis or cisplatin exposure: a case report

**DOI:** 10.1186/s13256-024-04515-1

**Published:** 2024-05-11

**Authors:** Chirag Mehta, Fatima Raza

**Affiliations:** 1 The Warren Alpert Medical School of Brown University, Providence, USA; 2 The Norman and Rosalie Fain Health Centers, 164 Summit Ave, Providence, RI 02906 USA

**Keywords:** *In situ*, Aortic thrombus, Hypercoagulability of malignancy, Lung adenocarcinoma with metastases, Case report

## Abstract

**Background:**

An ascending aortic thrombus is exceedingly rare. Two instances have been reported in the setting of lung cancer, but only after cisplatin use, which is associated with hypercoagulability. We present the first case of a patient with lung cancer who developed an ascending aortic thrombus without structural risk factors or chemotherapy use.

**Case:**

A 60-year-old white female with significant smoking history presented with several weeks of malaise. A chest computed tomography scan revealed a 2.2-cm right upper lobe mass. As an outpatient, right hilar lymph node immunohistochemistry (IHC) samples via endobronchial ultrasound confirmed thyroid transcription factor-1 adenocarcinoma. After the procedure, the patient endorsed dyspnea and was advised to go to the emergency department. A chest computed tomography angiography identified a new 2.4 × 1.1 × 1.1 cm thrombus within the proximal aortic arch. No pulmonary emboli or intrapulmonary shunts were identified. A hypercoagulable workup was negative. Transthoracic echocardiogram was without left ventricular thrombus, akinesis or hypokinesis, left atrial dilation, or intracardiac shunts. A lower extremity ultrasound was negative for deep vein thrombosis. Given the procedural risk, thrombectomy was deferred. The patient was transitioned to enoxaparin, and a repeat computed tomography for resolution is in process.

**Conclusion:**

To our knowledge, this is the only case detailing an *in situ* ascending aortic thrombus in the setting of lung cancer, without structural risk factors, chemotherapy use, or other hypercoagulable comorbidities. Optimal management for an aortic thrombus and malignant disease is less clear. Clinicians should be vigilant for unusual arterial thromboses in patients with high metastatic burden.

## Learning objectives

- An ascending aortic thrombus without structural risk factors is very rare owing to high laminar flow which precludes hemostasis.
- Management of an ascending aortic thrombus is unclear and should be individualized; however, salvage therapy is reasonable.
- Unusual arterial thromboses should be considered in patients with florid malignancy.

## Introduction

An ascending aortic thrombus without structural risk factors, such as aneurysm, dissection, aortitis, trauma, or severe atherosclerosis, is exceedingly rare. There are only a handful of case reports detailing aortic thrombosis in the context of malignant disease, but the majority involve the thoracic or descending abdominal aorta. The few reports that do include the ascending aorta occurred in patients following cisplatin usage, which is associated with a prothrombotic state. We present the first reported case of a patient who developed an *in situ* ascending aortic thrombus in the setting of lung malignancy who had not undergone chemotherapy and had no previous aortic structural risk factors.

## Case presentation

**Fig. 1:**
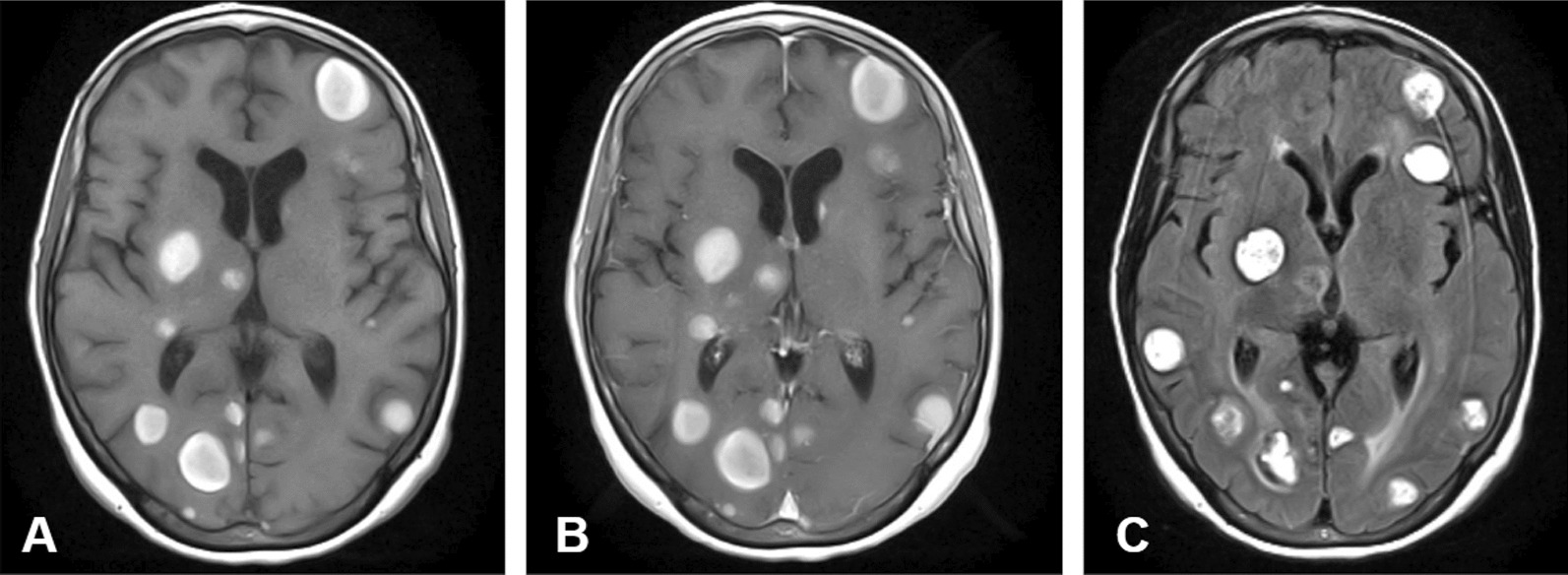
Magnetic resonance image brain axial slices at the level of the hypothalamus demonstrating innumerable round, bilateral lesions consistent with florid metastatic spread. **A** T1-weighted without contrast. **B** T1-weighted with contrast. **C** T2-fluid attenuated inversion recovery imaging demonstrating vasogenic edema of occipital lesions

**Fig. 2:**
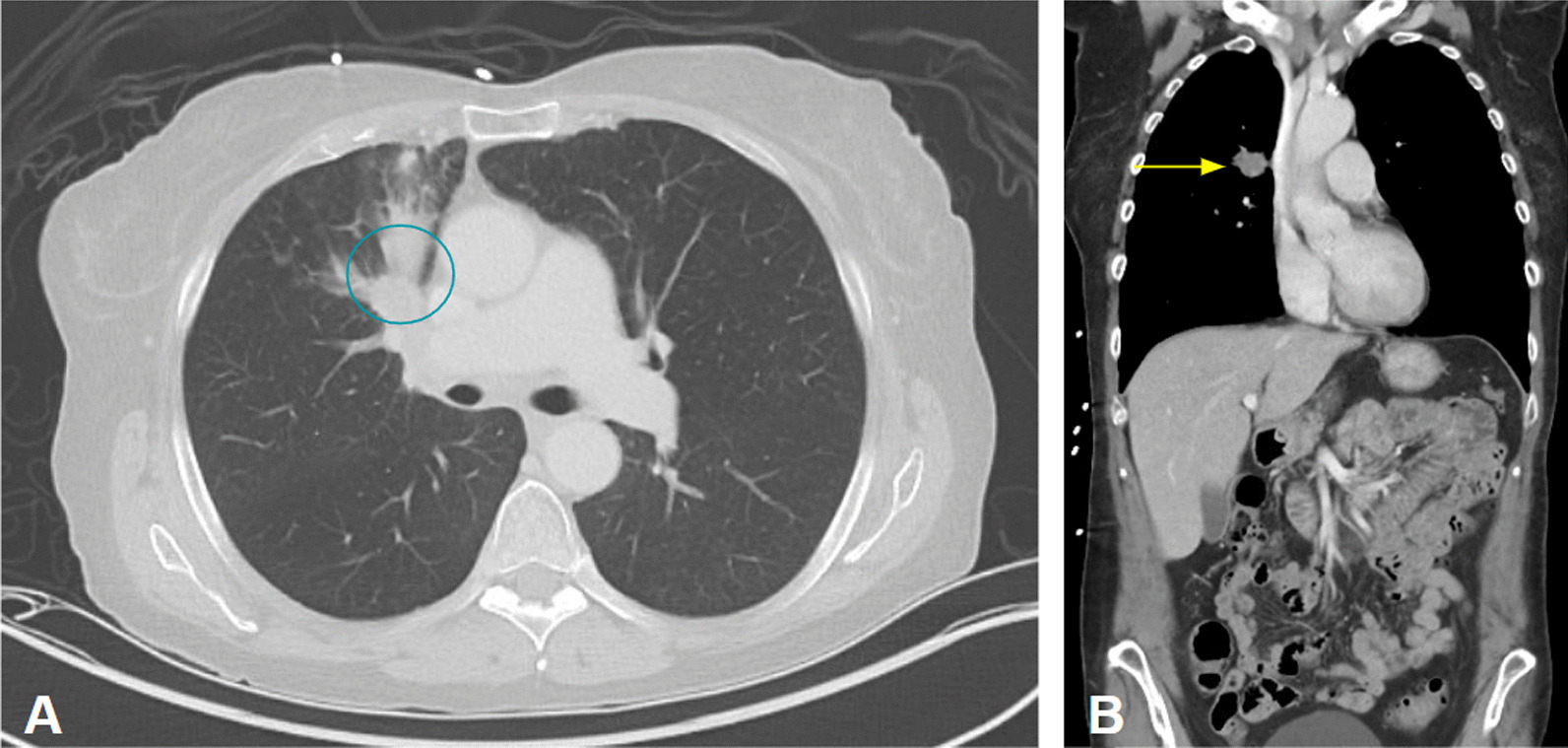
Axial (**A**) and coronal (**B**) slices of the initial contrast-enhanced chest computed tomography demonstrating a 2.2 × 2.0 cm right upper lobe nodule (blue circle and yellow arrow) with distal scattered nodular opacities

**Fig. 3:**
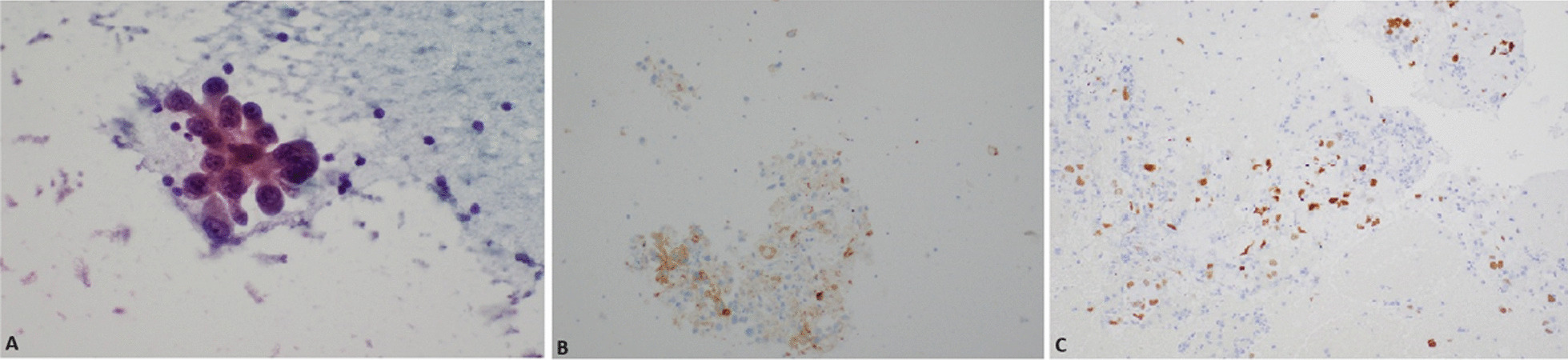
Histopathology of fine needle aspirate from station 5 hilar lymph node, obtainable via endobronchial ultrasound. **A** Papanicolaou staining showing positivity for adenocarcinoma. **B** monoclonal antibody (8G7G3/1) against thyroid transcription factor 1 demonstrating positive nuclear expression (brown staining). **C** Monoclonal antibody (Ventana-SP263) against programmed death-ligand 1 showing < 30% of tumor cells with positive expression (brown staining) to gauge candidacy for pembrolizumab therapy

**Fig. 4:**
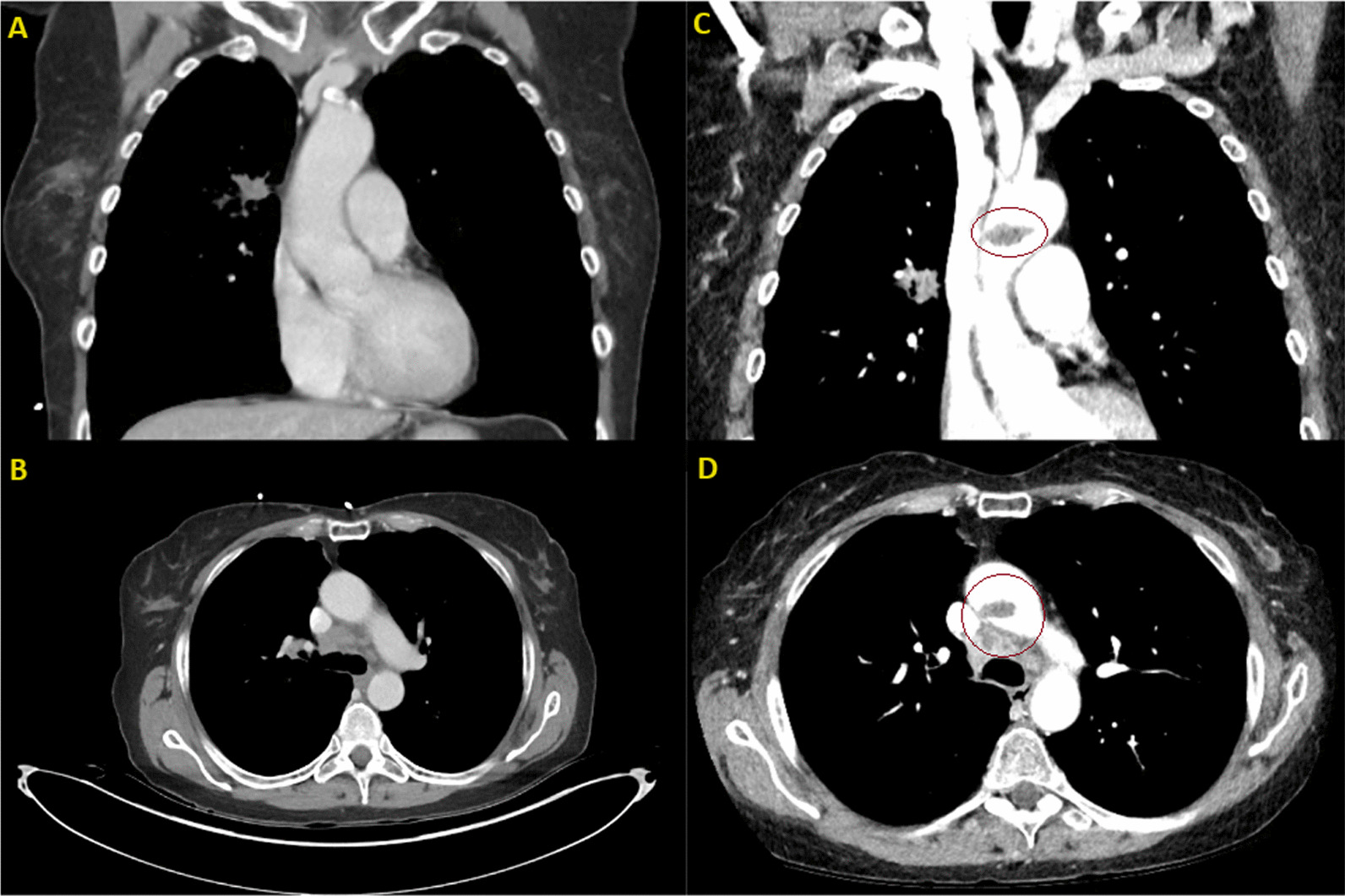
**A** and **B** Coronal and axial sections of the initial contrast enhanced computed tomography of the chest, respectively (from Fig. 2), failing to demonstrate aortic pathology, including dissection or aneurysm. There is calcification noted at the ostia of the left common carotid and left subclavian, but negligent atherosclerosis in the ascending aorta. **C** and **D** Comparison of coronal and axial slices, respectively, this time from a computed tomography angiogram of the chest, demonstrating the new aortic thrombus proximal to the aortic arch

A 60-year-old white female with a 45-pack-year smoking history and history of asthma presented to the emergency department with several weeks of generalized, nonfocal headache, and dizziness. She denied any visual obscurations, nausea, or vomiting. The patient’s family history was only notable for maternal and paternal hypertension and type 2 diabetes mellitus, but no hypercoagulable diseases. A magnetic resonance image (MRI) of the brain with and without contrast demonstrated innumerable round, bilateral lesions with peripheral hyperdensity suspicious for metastatic disease with internal hemorrhage but no midline shift (Fig. 1). A contrast-enhanced computed tomography (CT) of the chest revealed a 2.2-cm right upper lobe spiculated mass and extensive hilar lymphadenopathy, suggestive of a primary lung malignancy with metastatic adenopathy (Fig. 2). She decided to leave, against medical advice, but agreed to follow-up in the outpatient setting; she was given dexamethasone 4 mg every 6 h to take at home. At 2 weeks later in the pulmonary clinic, immunohistochemistry of samples from the right hilar lymph node obtained via endobronchial ultrasound confirmed thyroid transcription factor-1 (*TTF-1*) + adenocarcinoma and low positive programed death ligand 1 (*PD-L1*) at 30% (Fig. 3). Gene amplification unveiled driver mutations in Kirsten rat sarcoma viral oncogene homolog (*KRAS*) G12C and EIF1AZ. These findings all but confirmed stage IV lung adenocarcinoma. During the procedure, the patient became dyspneic and mildly anxious. Her vitals at the time were notable for sinus tachycardia with a heart rate (HR) of 101 bpm and mild hypoxia saturating 93% on room air. She denied any chest pain, pressure, cough, radiating pain to the left upper extremity, jaw pain, back pain, or sense of impending doom. The pulmonary team sent the patient to the emergency department (ED) for further evaluation. In the ED, the patient’s symptoms had improved. Her vital signs and physical exam findings were within normal limits. A respiratory pathogen panel, which included severe acute respiratory syndrome coronavirus 2 (SARS-CoV-2), was negative. A CT angiogram of the chest identified a new 2.4 × 1.1 × 1.1 cm thrombus within the proximal aortic arch (Fig. 4), but no pulmonary emboli or intrapulmonary right to left shunts were identified. A hypercoagulable workup was negative at both presentation and at 4 week follow-up (Table 1). The patient was promptly heparinized and carefully watched for symptoms of stroke, limb, or abdominal ischemia. A transthoracic echocardiogram with Definity contrast enhancement noted an left ventricular ejection fraction (LVEF) of 65%, but failed to show an LV thrombus, areas of LV akinesis or hypokinesis, left atrium (LA) dilation, or any atrial septal defects (ASDs), ventricular septal defects (VSDs), or patent foramen ovale (PFO) via agitated saline. An ultrasound of the lower extremities was similarly negative for deep vein thrombosis. Given the considerable procedural risk and skepticism about recurrence, surgical thrombectomy was deferred. The patient was transitioned to 1 mg/kg twice daily enoxaparin dosing. She remains asymptomatic and a repeat CT of her chest to gauge resolution is in process. She has undergone 2 weeks of whole brain radiation therapy and is pending initiation chemoimmunotherapy with carboplatin, pemetrexed, and pembrolizumab.

**Table 1:** Full hypercoagulable workup panel demonstrating that all values are within normal limits, suggesting that the ascending aortic thrombus was most likely due to hypercoagulability of malignancy

Coagulopathy	Reference range	Patient value
Antithrombin III	80–130%	108%
Factor V Leiden	1601 G > A	Negative for mutation
Prothrombin 20210	97 G > A	Negative for mutation
Lupus anticoagulant ratio	< 1.2	1.08
Anti-β2-glycoprotein	IgG: 0–20.0 U/mL IgM: 0–20.0 U/mL	IgG: < 6.4 U/mL IgM: < 1.1 U/mL
Anti-cardiolipin	IgG: < 15 GPL IgM: < 12.5 GPL	IgG: < 9.4 GPL IgM: < 9.4 GPL
Protein C	80–170%	> 140%
Protein S	60–140%	68%
Chronic DIC	Fibrinogen: 150–450 mg/dL PT and INR: 10.0–13.0 seconds and 0.8–1.2 PTT: 24.0–37.0 seconds	Fibrinogen: 448 mg/dL PT and INR: 11.2 seconds and 0.9 PTT: 37.0 seconds
Serum homocysteine	5–13.9 µmol/L	9.0 µmol/L
Methylenetetrahydrofolate reductase (MTHFR)	665C > T 1886A > C	Negative for both polymorphisms

*IgG* immunoglobulin G, *IgM* immunoglobulin M, *PT* prothrombin time, *INR* international normalized ratio, *PTT* partial thromboplastin time

## Discussion

It is well understood that malignancy can promote a state of hypercoagulability through an increase in procoagulant mucins and tissue factor along with the release of prothrombotic cytokines. An *in situ* ascending aortic thrombus in the absence of aortic substrate (i.e., dissection, aneurysm, trauma, aortitis, or aortic atherosclerosis) is an extremely rare occurrence, since high laminar flow within this area precludes hemostasis. In this case, the patient had no personal history nor family history of hypercoagulability. Moreover, she had a comprehensive hypercoagulable panel both on presentation and on follow-up, which was negative. As such, evidence points to her florid metastatic spread as the most compelling driver for her aortic thrombosis.

There are only a handful of case reports that detail aortic thrombosis due to hypercoagulability of malignancy [1–15], and within these studies, the majority have occurred in the descending thoracic or abdominal aorta following viscous energy dissipation, and only four cases involve the ascending aorta (Table 2). Of the four cases, two involved primary bronchogenic carcinomas, and both patients had undergone cisplatin chemotherapy, which is believed to promote a prothrombotic state from elevation of von Willebrand factor levels, hypomagnesemia-associated vasospasm, and LV dysfunction [18]. Optimal management for an aortic thrombus and malignant disease is not well established. Several studies support a conservative approach given periprocedural complications, risk for embolism with thrombus manipulation, and poor overall prognosis. In this case, our surgical team deferred intervention for similar reasons. However, it is reasonable to pursue an urgent salvage surgery to rescue ischemic limbs and visceral organs.

**Table 2:** Summary of published reports detailing aortic thromboses stratified by anatomic location in patients with underlying malignancy

Authors	Year	Patient	Malignancy	Thrombus location	Treatment
Farah *et al*. [1]	1993	74 F	Colon adenocarcinoma	Ascending aorta	UFH
Hahn *et al*. [2]	2011	74 M	Lung adenocarcinoma	Ascending aorta	LMWH
Mosquera *et al*. [3]	2009	56 F	Lung epidermoid carcinoma	Ascending aorta	Thrombectomy
Sato *et al*. [4]	2022	63 M	Esophageal carcinoma	Ascending aorta	Thrombectomy
Chin *et al*. [5]	2010	50 M	Small cell lung cancer	Aortic arch	LMWH
Kim *et al*. [6]	2016	72 M	Pancreatic adenocarcinoma	Descending aorta	UFH
		60 F	Breast carcinoma	Descending aorta	UFH
Mark *et al*. [7]	2005	57 M	Undifferentiated adenocarcinoma	Descending aorta	Axillobifem bypass
Yagyu *et al*. [8]	2019	70 M	Gastric adenocarcinoma	Descending aorta	UFH
Boon *et al*. [9]	2016	70 M	Gastric adenocarcinoma	Abdominal aorta	LMWH
Kim *et al*. [6]	2016	55 M	Pancreatic adenocarcinoma	Abdominal aorta	UFH
		44 F	Gastric adenocarcinoma	Abdominal aorta	Thrombectomy
Dieckmann *et al*. [10]	2009	49 M	Testicular seminoma	Abdominal aorta	LMWH
Serck *et al*. [11]	2009	59 F	Sigmoid colon adenocarcinoma	Abdominal aorta	UFH
Faisham *et al*. [12]	2005	47 F	Undifferentiated sarcoma	Abdominal aorta	UFH
Poiree *et al*. [13]	2004	45 F	Pancreatic adenocarcinoma	Abdominal aorta	UFH
		53 M	T cell lymphoma	Abdominal aorta	–-
Casillas *et al*. [14]	2002	68 F	Rectal adenocarcinoma	Abdominal aorta	Axillobifem bypass
Kawachi *et al*. [15]	1996	44 M	Non-Hodgkin’s lymphoma	Abdominal aorta	Thrombectomy
Leong *et al*. [16]	1995	38 M	Leukemia	Abdominal aorta	Warfarin
Arima *et al*. [17]	2019	54 f	Sigmoid colon adenocarcinoma	Abdominal aorta	UFH

*UFH* unfractionated heparin, *LMWH* low-molecular-weight heparin

## Conclusion

To our knowledge, this is the only case detailing an *in situ* ascending aortic thrombus in the setting of lung cancer without structural risk factors, chemotherapy use, or other hypercoagulable comorbidities. Optimal management for an aortic thrombus and malignant disease is less clear. Clinicians should be vigilant for unusual arterial thromboses in patients with high metastatic burden.

## Data Availability

Ancillary data can be provided upon author request.
